# Rapid changes in arousal states of healthy volunteers during robot-assisted gait training: a quantitative time-series electroencephalography study

**DOI:** 10.1186/1743-0003-11-59

**Published:** 2014-04-12

**Authors:** Yoshie Nakanishi, Futoshi Wada, Satoru Saeki, Kenji Hachisuka

**Affiliations:** 1 Department of Rehabilitation Medicine, Faculty of Medicine, University of Occupational and Environmental Health, Japan, 1-1, Iseigaoka, Yahatanishi-ku, Kitakyushu 807-8555, Japan; 2 Department of Rehabilitation, Wakamatsu Hospital of the University of Occupational and Environmental Health, Japan, 1-1 Hama Machi, Wakamatsu-ku, Kitakyushu 808-0024, Japan

**Keywords:** Robot-assisted gait training, Arousal state, Electroencephalography, Warning sound stimulation, Verbal stimulation

## Abstract

**Background:**

Robot-assisted gait training (RAGT) is expected to be an effective rehabilitative intervention for patients with gait disturbances. However, the monotonous gait pattern provided by robotic guidance tends to induce sleepiness, and the resultant decreased arousal during RAGT may negatively affect gait training progress. This study assessed electroencephalography (EEG)-based, objective sleepiness during RAGT and examined whether verbal or nonverbal warning sounds are effective stimuli for counteracting such sleepiness.

**Methods:**

Twelve healthy men walked on a treadmill for 6 min, while being guided by a Gait-Assistance Robot, under 3 experimental conditions: with sine-wave sound stimulation (SS), verbal sound stimulation (VS), and no sound stimulation (NS). The volunteers were provided with warning sound stimulation at 4 min (ST1), 4 min 20 s (ST2), 4 min 40 s (ST3), and 5 min (ST4) after the start of RAGT. EEGs were recorded at the central (Cz) and occipital (O1 and O2) regions (International 10–20 system) before and during RAGT, and 4-s segments of EEG data were extracted from the filtered data during the 8 experimental periods: middle of the eyes-closed condition; middle of the eyes-open condition; beginning of RAGT; immediately before ST1; immediately after ST1, ST2, ST3, and ST4. According to the method used in the Karolinska drowsiness test, the power densities of the theta, alpha 1, and alpha 2 bands were calculated as indices of objective sleepiness.

**Results:**

Comparisons of the findings between baseline and before ST showed that the power densities of the alpha 1 and 2 bands tended to increase, whereas the theta power density increased significantly (*P* < .05). During NS, the power densities remained at a constant high level until after ST4. During SS and VS, the power densities were attenuated immediately to the same degree and maintained at a constant low level until after ST4.

**Conclusions:**

This study is the first to demonstrate that EEG-measured arousal levels decrease within a short time during RAGT, but are restored and maintained by intermittent warning sound stimulation.

## Background

Stroke is a leading cause of death and a major contributor to adult neurologic disabilities, including gait disturbances, which are considered among the most devastating daily activity disabilities. Therefore, alleviation of gait disturbances remains a major component of post-stroke rehabilitation. To restore gait function, modern concepts of rehabilitation favor a repetitive, task-specific approach [1]. Furthermore, in recent years, higher intensities and repetitions of walking practices have been reported to result in better outcomes in post-stroke patients [2,3].

Robot-assisted gait training (RAGT) is a method used to provide adequate gait training to patients, in addition to overground gait training [4] and treadmill gait training, with and without partial body weight support [5]. A few electromechanical devices for RAGT are commercially available. Lokomat (Hocoma, Zurich, Switzerland) is an exoskeleton-driven gait orthosis consisting of frames and servomotors positioned over a patient’s lower extremities, a harness for suspension of body weight, and a treadmill [6]. Gait Trainer (Reha-Stim, Berlin, Germany) is an end-effector device, based on a double crank and rocker gear system, consisting of 2 foot plates, 2 bars, 2 rockers, and 2 cranks. The patient is suspended by a harness and stands on the 2 foot plates, which symmetrically simulate the stance and swing phases of walking [7]. The Gait-Assistance Robot (GAR) is a prototype gait-training robot developed by Yaskawa Electric Corporation (Kitakyushu, Japan) [8,9]. GAR features 4 robotic arms that automatically and separately control both lower extremities, the capacity for patients to walk on a treadmill with their full body weight on both lower extremities, and a visual foot pressure biofeedback system. Despite the availability of these advanced rehabilitation systems, whether RAGT devices facilitate or enhance the recovery of gait function better than conventional overground gait training (COGT) remains unclear [10]. In recent years, several randomized controlled trials have compared RAGT with COGT, showing that the outcomes of RAGT remain inconsistent [11-14]. Hidler et al. [11] proposed several reasons for RAGT being less effective than COGT in facilitating recovery of walking ability, including the possibility that "robotic guidance" might reduce volitional muscle activity and subsequent learning. Moreover, patients’ effort might not be at the highest level during training sessions because of too much assistance being provided by the robotic device. If RAGT is monotonous and less volitional, patients may get bored and sleepy during training sessions, as has been observed during monotonous driving tasks [15]. In fact, we have encountered patients who feel sleepy during RAGT. We often provide verbal encouragement to these patients to dispel their sleepiness and enhance their motor learning, based on the therapeutic process of rehabilitation [16]. Thus, patients must maintain their effort at the highest level and refrain from becoming drowsy during RAGT.

Warning sound stimulation is an effective method for focusing patients’ attention on a task and preventing sleepiness. In sleep-related research, warning sounds have been widely used as external stimuli for sleepy or sleeping subjects [17,18]. Warning sound stimulation may be effective for maintaining patients’ attention or awakening sleepy patients during RAGT, but studies involving objective measures of arousal or sleepiness during RAGT have not been reported. Sound stimulation affects the arousal state of healthy adults, and the characteristics of sound stimulation, such as its intensity [19], schedule (continuous or intermittent) [20], and type [21], are important. Verbal stimuli, in the form of encouraging statements, are often used in the clinical setting to elicit the voluntary effort of patients. In several studies, verbal stimulation during a task induced higher performance than that induced by nonverbal stimulation [22-24].

Based on the background information and our clinical experience, we hypothesized that the arousal of patients during RAGT may be altered because of a monotonous training pattern and that verbal stimulation may be effective in maintaining patients’ attention and preventing sleepiness. Thus, the aim of this study was to determine whether (1) the arousal state of healthy volunteers could be changed at an early period during RAGT, (2) the arousal state during RAGT could be affected by intermittent warning sound stimulation, and (3) whether verbal stimulation is superior to nonverbal stimulation for influencing electroencephalography (EEG)-measured arousal states during RAGT.

## Methods

### Participants

The study participants included 12 healthy male volunteers (age, 39.3 ± 1.8 years; height, 168.4 ± 0.8 cm; weight, 64.9 ± 2.3 kg). These individuals did not have a history of neurologic, musculoskeletal, orthopedic diseases or otological disorder, and were not using psychotropic medications. The study was approved by the Ethics Committee at the University of Occupational and Environmental Health, Japan (approval number, 09–93), and all volunteers provided informed consent before participating in the study.

### Gait-Assistance Robot

**Figure 1:**
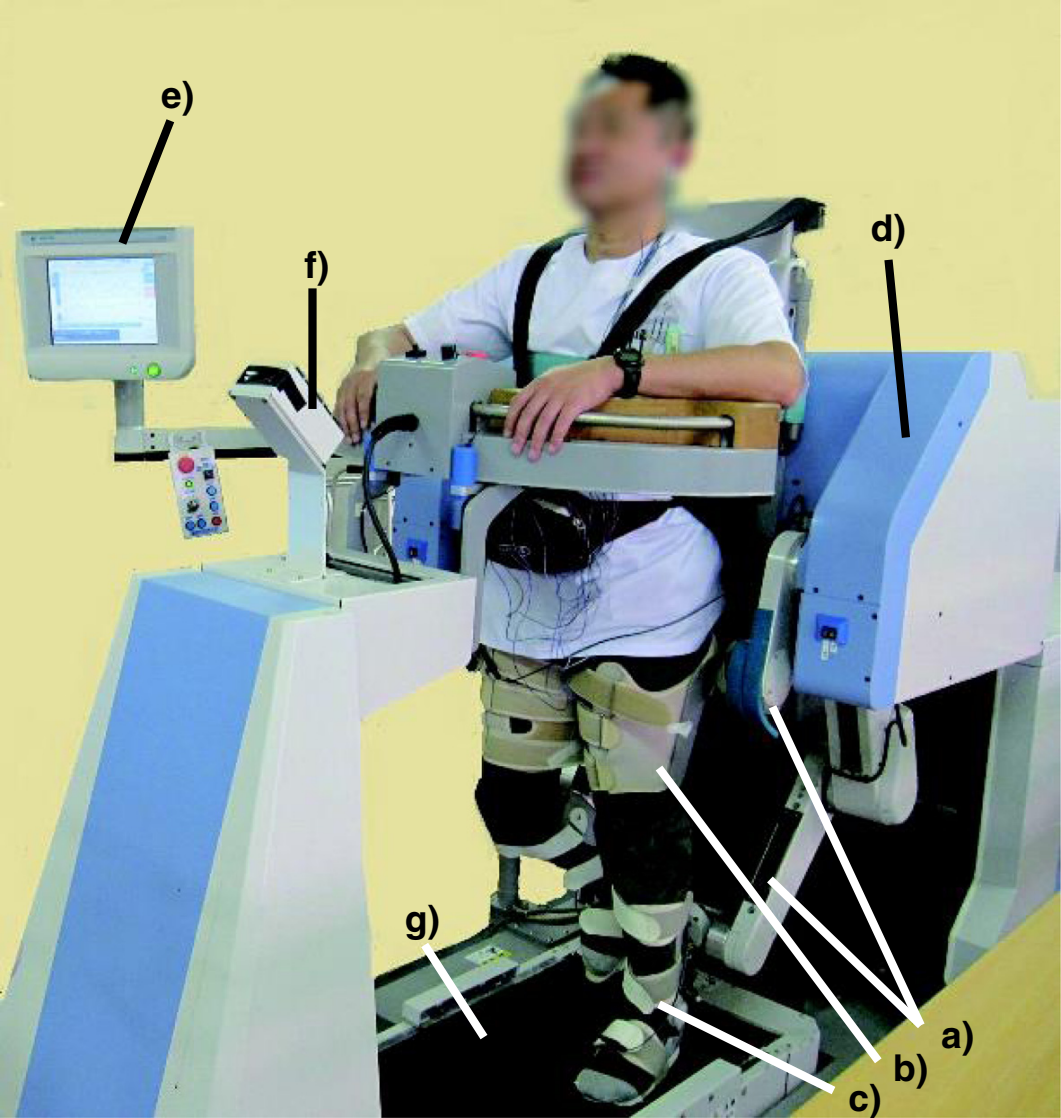
**Gait-Assistance Robot.** The Gait-Assistance Robot consists of **a)** 4 robotic arms for the thighs and lower legs, **b)** thigh cuffs, **c)** lower leg apparatuses, **d)** a generator, **e)** a control panel, **f)** lights for the foot pressure biofeedback system, and **g)** a treadmill.

The GAR was the robotic device used in this study (Figure 1). An additional movie file shows this in more detail [see Additional file 1]. The main features distinguishing GAR from the Lokomat and Gait Trainer include the 4 robotic arms, a full weight-bearing system, and a visual foot pressure biofeedback system. GAR has 3 exercise modes (active, passive, and active-assistive) and can be used for providing RAGT to acute and sub-acute stroke patients with severe gait disturbances. A randomized controlled study has reported the outcomes of RAGT with GAR in patients with severe gait disturbance (in preparation for submission).

### Types of warning sound stimulation

Three types of experimental conditions were investigated in this RAGT study, including sine-wave sound stimulation (SS) [25], verbal sound stimulation (VS), and no sound stimulation (NS). SS was produced by software on a personal computer, while VS was provided in the form of a single Japanese word, "ganbatte," which means "keep it up" in English. During NS, sound stimulation was not provided.

Before the experiment, we attempted to equalize the sound intensity and duration between SS and VS. Sound intensity was monitored as A-weighted sound pressure level (dBA) using a sound-level meter (Sound Level Meter NL-20, Rion, Tokyo, Japan). Sound duration was measured simultaneously using a digital sound editing application on a personal computer. First, 5 trials of VS were conducted, in which the examiner spoke with consistent intonation and inflection. The average sound intensities and durations of the 5 trials were 82.0 ± 2.4 dBA and 2.0 ± 0.1 s, respectively. On the basis of these values, the sound intensity and duration of SS were set at 82.0 dBA and 2.0 s, respectively.

### General experimental procedure

Each subject was instructed to sit in the GAR, and thigh cuffs and lower leg equipment were attached to the lower extremities. The GAR automatically changed the subject’s position from sitting to standing. Then, the subject was asked to walk and synchronize his movements with those of the robotic arms.

**Figure 2:**
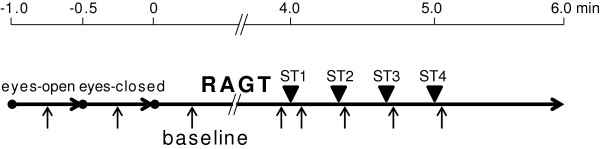
**Experimental protocol.** Each subject stood in the Gait-Assistance Robot (GAR) with closed eyes for 30 s and then with open eyes for 30 s. Subsequently, robot-assisted gait training (RAGT) proceeded for 6 min. Sine-wave sound stimulation (SS), verbal sound stimulation (VS), and no sound stimulation (NS) were provided at 4 min (ST1), 4 min 20 s (ST2), 4 min 40 s (ST3), and 5 min (ST4) after the start of RAGT. Electroencephalography (EEG) were recorded throughout the experimental protocol, and each 4-s segment of EEG data (arrow, ↑) was extracted from filtered data during the 8 experimental periods: middle of the eyes-closed condition; middle of the eyes-open condition; beginning of RAGT (baseline); immediately before ST1; immediately after ST1, ST2, ST3, and ST4. ▼ST1–ST4: first-fourth 2-s sound stimulation. ↑: 4-s segments of EEG data.

Figure 2 shows an outline of the experimental protocol. First, each participant, standing in the GAR, was asked to close his eyes for 30 s and then open them for 30 s. Subsequently, the participant underwent RAGT for 6 min on the treadmill at a speed of 0.11 m/s in active-assistive mode. SS, VS, and NS were provided at 4 min (ST1), 4 min 20 s (ST2), 4 min 40 s (ST3), and 5 min (ST4) after the start of RAGT. The order of the 3 stimulations was randomized, and the participants were not notified of the order. EEGs were recorded throughout the experimental protocol by an experienced EEG technician. During this experimental procedure, the GAR visual biofeedback system was switched off.

### EEG recording

A portable bio-signal recorder (Polymate II AP216, TEAC, Tokyo, Japan) was used to record EEGs. Active electrodes were attached to the central (Cz) and occipital (O1 and O2) scalp sites (International 10–20 system), referenced to a linked earlobe (A1-A2). The sampling rate for all channels was 1000 Hz, and the time constant was 0.3 s. Electrode impedance was maintained below 10 kΩ. A ground terminal was connected between the personal computer and the ground to eliminate noise from the alternating current power. Vertical and horizontal electro-oculograms were recorded to monitor artifacts caused by eye blinks and movements. Unlike during walking on the floor, the volunteers did not move their bodies and heads while walking on the GAR treadmill, because the GAR controlled their lower extremities and loosely restrained the trunk. Therefore, EEG noise was not contaminated by body movements and did not influence the results of the experiment. An additional movie file shows this in more detail [see Additional file 2].

All raw EEG data were filtered with a high frequency cut-off at 50 Hz using a wave viewer program (Vital Tracer, Kissei Comtec, Matsumoto, Japan), and then transferred to a multi-bioinformation analyzer program (Bimutas II, Kissei Comtec) for further data processing. Distinct artifacts in the EEGs were excluded by visual inspection.

Each 4-s segment of EEG data was extracted from the filtered data during the 8 experimental periods: middle of the eyes-closed condition; middle of the eyes-open condition; beginning of RAGT (baseline); immediately before ST1; immediately after ST1, ST2, ST3, and ST4 [26,27]. The power density of each segment was calculated using fast Fourier transform with a Hamming window [26,27]. Absolute power density (μV^2^) was divided into 5 frequency bands: delta (2.0–3.9 Hz), theta (4.0–7.9 Hz), alpha 1 (8.0–9.9 Hz), alpha 2 (10.0–12.9 Hz), and beta (13.0–30.0 Hz). The Cz values represented the power density at the central region, and the mean values at O1 and O2 represented the power density at the occipital region.

### EEG measures during the eyes-closed and eyes-open conditions

The EEG data were promptly inspected by the EEG technician when the participant’s eyes were open or closed. In normal adults, alpha activity is dominant when the eyes are closed (alpha rhythm) and is attenuated when the eyes are open (alpha attenuation) in the occipital region. Confirmation of the obvious alpha rhythm and its attenuation in the EEG raw data of each participant was necessary before the warning sound stimulation because objective sleepiness in this study was evaluated based on EEG changes [28]. Therefore, the absolute alpha power densities (8.0–12.9 Hz) in the occipital region during the eyes-closed and eyes-open conditions were analyzed, and the relative alpha power densities were calculated as a percentage of the absolute power density of the alpha band (8.0–12.9 Hz) relative to the absolute power density of the global electroencephalography band (2.0–30.0 Hz).

### Estimation of objective sleepiness

The EEG-measured theta and alpha power densities in the eyes-open condition were evaluated as an index of objective sleepiness. EEGs were recorded using a method similar to that used in the Karolinska drowsiness test [29], based on the principle that the proportion of theta and alpha activity increases from wakefulness to sleepiness in the eyes-open condition [29-32]. During EEG measurements, each subject was instructed to relax with his eyes open, and to focus on a 5-cm dot attached to a wall 1.5 m away, while avoiding head movement.

### Statistical analysis

All values are shown as means ± SE. Differences in alpha power densities between the eyes-closed and eyes-open conditions were analyzed using paired *t*-tests. Theta and alpha power densities were compared among 6 segments (baseline, before ST1, after ST1, after ST2, after ST3, and after ST4) and 3 types of stimulation using 2-way repeated measures analysis of variance. Dunnett’s post-hoc test was conducted to identify each variable between baseline and the subsequent 5 segments. Bonferroni’s multiple comparison was conducted to clarify the differences among the 3 stimulations.

SPSS version 19 software (IBM, Tokyo, Japan) was used for statistical analysis and a *P* value < .05 was considered significant.

## Results

### Differences in EEG data between eyes-closed and eyes-open conditions

Table 1 shows the absolute and relative alpha power densities in the occipital region. During the eyes-closed condition, alpha activity occupied 60.8% of the global EEG bands in the occipital region. Absolute and relative alpha power densities were significantly lower during the eyes-open condition than during the eyes-closed condition (*P* < .0001).

**Table 1:** Absolute and relative alpha power densities in the occipital region

**Absolute power density (mean ± SE, μV**^**2**^**)**		**Relative power density**^**a **^**(mean ± SE, %)**	
**Eyes closed**	**Eyes open**	**Eyes closed**	**Eyes open**
23.9 ± 3.5*	2.4 ± 0.2	60.8 ± 2.4*	26.0 ± 1.7

*paired t-test, eyes closed vs. eyes open, *P* < .0001.

^a^Relative power density = {absolute power density of the alpha band (8.0–12.9 Hz)}/{absolute power density of the global EEG band (2.0–30.0 Hz)} × 100 (%).

### EEG-measured arousal without warning sound stimulation

**Figure 3:**
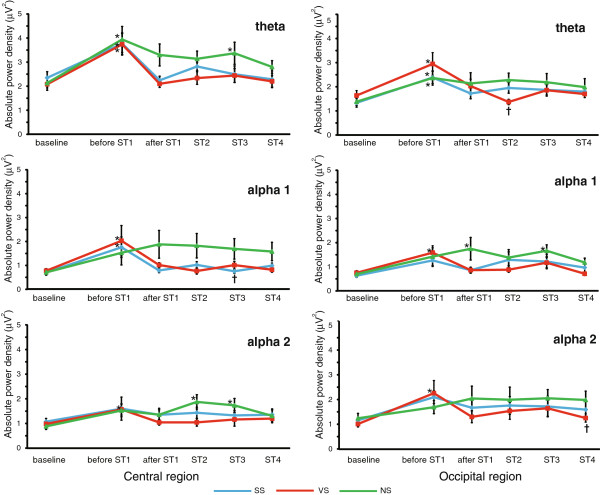
**Absolute power densities of the theta, alpha 1, and alpha 2 bands.** Blue, red, and green lines indicate the power densities of each wave activity under sine-wave sound stimulation (SS), verbal sound stimulation (VS), and no sound stimulation (NS), respectively. There were significant differences in the power densities between baseline and the other segments (*P < .05, Dunnett’s test) and among the 3 types of stimulation (^†^*P* < .05, Bonferroni multiple comparison).

Figure 3 shows the absolute power densities of the theta, alpha 1, and alpha 2 bands of the central and occipital regions during RAGT. During NS, the absolute theta power density increased significantly in the central and occipital regions before ST1 compared to baseline (*P* < .015), and these increments were significantly or relatively maintained until after ST4 (after ST3 in the central region, *P <* .01). The absolute power densities of the alpha 1 and 2 bands were significantly or relatively increased compared to baseline (alpha 1, after ST1 and 3 in the occipital region, *P* < .05; alpha 2, after ST2 and 3 in the central region, *P* < .05).

### Effect of warning sound stimulation on EEG-measured arousal state

**Figure 4:**
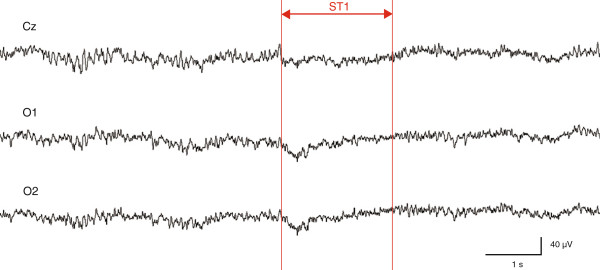
**A representative example of raw electroencephalography data around ST1.** Decreases in slow wave activity were observed after ST1 in the central (Cz) and occipital (O1 and O2) regions.

Figure 4 shows a representative example of raw EEG data around ST1. An immediate decrease in slow wave activity was observed after warning sound stimulation in the Cz, O1, and O2 regions.

**Figure 5:**
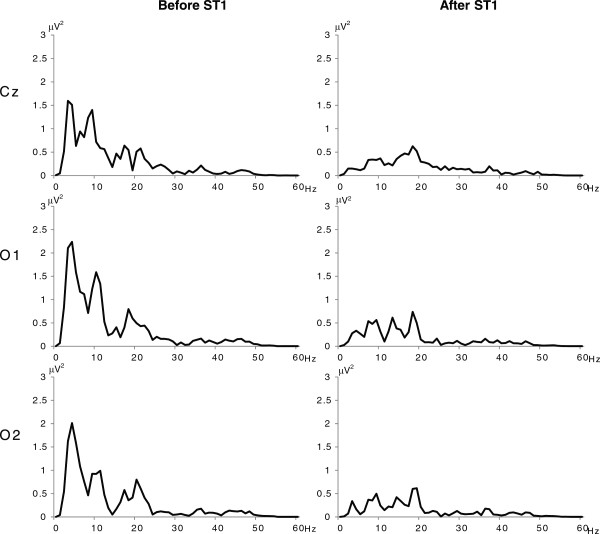
**A representative example of power spectral density at Cz, O1, and O2.** Power spectral densities of EEG data shows plenty of theta and alpha activities 4 minutes after the start of RAGT (Before ST1), and almost absent theta activities and moderate decreases in alpha activities immediately after warning sound stimulation (After ST1).

Figure 5 shows a representative example of the power spectral density at Cz, O1, and O2. Almost absent theta activities and moderate decreases in alpha activities were observed due to awakeness after ST1.

Figure 3 shows the influence on the absolute power densities of warning sound stimulation. During SS and VS, the absolute theta power density significantly increased before ST1 (*P* < .001) in both the central and occipital regions. However, it immediately decreased after ST1 and tended to remain at a baseline level until after ST4. There were no significant differences between the baseline level and that observed between after ST1 to after ST4. The absolute power densities of both the alpha 1 and 2 bands were significantly and relatively increased before ST (during SS, alpha 1 in the central region, *P* < .05; during VS, alpha 1 and alpha 2 in the central and occipital regions, *P* < .05). These increments promptly decreased after ST1 and were maintained at a baseline level, thereafter; there were no significant differences between the baseline values and those recorded from after ST1 to after ST4.

### Differences in sound stimulation types

Figure 3 shows the relationships between the absolute power densities and the stimulation types. At baseline and before ST1, there were no significant differences among the 3 types of stimulation (*P* > .1). From after ST1 to after ST4, all mean power densities during SS and VS were significantly or relatively lower than those during NS (during SS, alpha 1 after ST3 in the central region, *P* < .05; during VS, theta after ST2 and alpha 2 after ST4 in the occipital region, *P* < .05). There were no significant differences in the power densities between SS and VS.

## Discussion

To our knowledge, this is the first study to assess arousal states during RAGT using EEG. The major findings of this study were that arousal level decreased significantly during RAGT in healthy volunteers and that warning sound stimulation restored the decreased arousal state to its original level. There was no difference in the arousal level obtained between verbal and nonverbal stimulation.

### EEG-measured arousal state during RAGT

In this study, EEGs were recorded as objective indices of sleepiness during RAGT. EEG measures are considered to detect the changes from wakefulness to light sleepiness, which would not be observed using self-reports [33]. Without any sound stimulation, the power densities of the theta, alpha 1, and alpha 2 bands increased within 4 min of the beginning of RAGT. Increments in theta and alpha activity during the eyes-open condition are assumed to be a reliable index of objective sleepiness [30-32]. Therefore, the increments of theta and alpha activities observed in this study indicate that RAGT induced sleepiness. This result is compatible with our clinical observations that many patients report sleepiness during RAGT. Monotony is generally recognized as a major factor causing boredom, which induces sleepiness during a task, e.g., driver sleepiness is more likely to occur on monotonous motorways [15,32,34]. The robotic arms of the GAR guide the patients’ lower limbs through the same trajectory patterns at the same frequency; thus, RAGT may be a monotonous task that causes boredom.

Surprisingly, arousal levels started decreasing within only 4 min after the start of RAGT. This rapid decrease in arousal level was also reported by Laure et al. [35] for another monotonous task, taking a computer-based sustained attention task. They reported increased sleepiness and decreased performance in their participants within only 4.5 min after beginning the task. RAGT may be monotonous enough to induce more sleepiness than originally expected.

In our results, the increase in theta activity was greater than the increase in alpha activity during RAGT. In general, theta activity is mainly observed in the stage between drowsiness and light sleep, whereas alpha activity is mainly seen in the relaxed state [29,36]. According to the changes in theta and alpha activities, we estimate that the arousal state during RAGT was not relaxation, but drowsiness. Indeed, the arousal level appreciably decreased during RAGT. In this study, RAGT was performed for only 6 min, whereas RAGT for a hemiplegic patient is usually performed for more than 20 min; therefore, a patient’s arousal state during RAGT may decline to an even lower level. Such arousal states may interrupt voluntary effort during RAGT and consequently diminish the effectiveness of training. Numerous studies have reported a relationship between sleepiness and performance in prolonged monotonous tasks; for example, monotonous driving has been shown to induce sleepiness and impair driving performance [32,37]. The rapidly increasing sleepiness during RAGT may also interrupt a patient’s voluntary efforts and diminish the clinical benefits. Therefore, sufficient stimulation is required to arouse patients during the early stages of RAGT.

In this study, each 4-s segment of EEG data was extracted and analyzed using fast Fourier transform with a Hamming window. However, fast Fourier transform has limited temporal resolution, and it may be impossible to detect slight, quick changes occurring during the 4-s intervals. In future studies, we would like to examine the use of time frequency analyses, such as wavelet transformation, to detect immediate responses.

### Effect of warning sound stimulation

The decreased arousal level during RAGT was promptly restored by either SS or VS. A study by Landström et al. [18] used EEG to confirm that unpredictable sound has an arousing effect and prevents drowsiness; sound enhances objective wakefulness [18]. Therefore, auditory stimulation may be a method for preventing sleepiness during RAGT. In addition, an increased arousal state could be maintained by providing consecutive and intermittent sounds during RAGT. Continuous and monotonous sounds (e.g., white noise and wave sound) generally induce sleep, whereas intermittent sounds maintain arousal [18,38,39]. Landström et al. [18] reported that wakefulness, enhanced by unexpected sounds, was maintained by intermittent sound. Thus, intermittent sound stimulation may be effective in maintaining arousal during RAGT.

### Differences in sound stimulation types

In our study, SS increased patient arousal levels to approximately the same degree as VS. This phenomenon is supported by the arousal state being strongly influenced by sound intensity. The sound pressure levels of SS and VS (82.0 dBA) had sufficient intensity to increase subjects’ arousal. Studies on the effects of sound have reported that loud and unpredictable noises tend to increase arousal. Pearsons et al. [19] reviewed the findings of 21 studies that investigated the effects of noise on sleep. In their review, a strong correlation was found between noise levels and EEG-measured arousal states. Landström et al. [18] also found that unpredictable 70-dBA sound increased arousal levels and that intermittent 70-dBA sound maintained an aroused state. Although no differences in arousal levels were found between SS and VS at 82.0 dBA, in our experiment, differences in arousal levels, based on sound type, would become clear if the sound intensity were lower than the sound stimulation we used. Further studies on intensities, schedules, and types of warning sound stimuli are needed to investigate how to effectively maintain an aroused state during RAGT.

### Limitations

The current study has several limitations. First, RAGT is a clinical intervention for patients with gait disturbances, such as stroke. However, the subjects in our study were healthy volunteers. Additional experiments are required, using patients with gait disturbances. Second, although RAGT, in the clinical setting, usually continues for 20–30 min, the present experimental RAGT conditions lasted for only 6 min. In future studies, we would like to investigate arousal changes during RAGT over longer periods.

## Conclusions

This study demonstrated that EEG-measured arousal in healthy persons quickly decreases during RAGT, but is restored and maintained by intermittent warning sound stimulation regardless of sound types. Further investigations are required to determine whether similar observations are made in an actual patient population with gait disturbance and also to determine the most effective encouragement.

## Competing interests

The authors declare that they have no competing interests.

## Authors’ contributions

YN participated in data collection, the design of the study, statistical analysis, and manuscript writing. FW contributed to the study design and coordination, and helped draft the manuscript. SS contributed to the study design and statistical analysis. KH oversaw the study design and coordination, contributed to interpretation of the findings, assisted with revision of the manuscript, obtained funding, and supervised the study. All authors read and approved the final manuscript.

## Supplementary Material

Additional file 1 **Movement of Gait-Assistance Robot (GAR).** The Gait-Assistance Robot consists of 4 robotic arms for the thighs and lower legs, thigh cuffs, lower leg apparatuses, a generator, a control panel, lights for the foot pressure biofeedback system, and a treadmill. The GAR enables full body weight-bearing while walking. Click here for file

Additional file 2 **Electroencephalography recording while robot-assisted gait training (RAGT).** Active electrodes were attached to the central and occipital scalp sites, referenced to a linked earlobe. Vertical and horizontal electro-oculograms were recorded to monitor artifacts caused by eye blinks and movements. The participant underwent RAGT for 6 min on the treadmill at a speed of 0.11 m/s in active-assistive mode. Click here for file
